# Harm From Foreign Body Ingestion in Adults and Children: A Systematic Review of Case Reports

**DOI:** 10.7759/cureus.95766

**Published:** 2025-10-30

**Authors:** Danielle Durant, Furqaan B Mohammed, Adrian Boyle

**Affiliations:** 1 Emergency Medicine, Addenbrooke's Hospital, Cambridge University Hospitals NHS Foundation Trust, Cambridge, GBR

**Keywords:** accidental, adult, blister pill pack, foreign bodies, foreign bodies in emergency medicine, foreign body, ingestion, intentional, paediatric, toothpick ingestion

## Abstract

Intentional and accidental foreign body ingestion is a common presentation seen in emergency medicine, with clinical characteristics and patterns in intentional and accidental foreign body ingestion in adult and paediatric populations being well documented in some foreign body groups, but not others. We conducted a systematic review of case reports and case series from EMBASE, Medline/PubMed, and CINAHL/Ebsco. Of 783 relevant papers, 199 were retrieved after screening, with 120 included in the final analysis. Cases involving magnets and batteries were excluded due to well-documented risks. A wide variety of harms were found, from a relatively small number of foreign body types, notably toothpicks, coins, medication blister packs, and fishbones. Characteristic patterns were found relating to age and type of foreign body ingested, and site of complications. Many injuries occurred distal to the diaphragm, not just above it. Fatal outcomes were described in both intentional and accidental ingestion. We concluded that foreign body ingestion can cause harm in multiple locations throughout the gastrointestinal tract. Clinical patterns vary with patient age and object characteristics. Passage beyond the diaphragm should not provide reassurance, as significant complications may still occur.

## Introduction and background

Foreign body ingestion is a common presentation to emergency departments, with objects such as coins and chicken bones being mentioned within the literature. Data from the United States show that the incidence of foreign body ingestion in adults has increased over the past two decades, from 3 to 5.3 per 100,000 persons. Hsieh et al. reported that between 1995 and 2017, 155,650 adult foreign body ingestions were recorded in the United States, of which 14% were intentional and the remaining 86% accidental [1].

Foreign body ingestion is more prevalent in children. Orsagh-Yentis et al. demonstrated that between 1995 and 2015, there were 759,074 recorded attendances to paediatric emergency departments for foreign body ingestion in the United States, with coins being the most common object [2], in contrast to the more diverse range of ingestions seen in adults [1].

The prevalence of foreign body ingestion is increasing, and the interventions and management of associated complications place a significant burden on healthcare systems. Reliable, reproducible data on the exact costs at a national or global level are limited. However, Chen et al. estimated the mean cost of managing foreign body ingestion in children in Canada to be $3469 Canadian dollars per patient [3]. When placed in the context of the numbers reported by Hsieh et al. [1] and Orsagh-Yentis et al. [2], this represents substantial annual expenditure on management, which is particularly relevant for national healthcare systems predominantly funded by taxpayers.

Certain foreign bodies, such as magnets, poisons, and batteries, are well recognised to cause significant harm and typically require intervention. The hazard profiles of these objects have been extensively described in the literature. Ingestion of two or more magnets can result in pressure necrosis, perforation, fistula formation, and death, as demonstrated in a systematic review by Xie et al. [4]. Batteries are associated with oesophageal mucosal injury, tracheo-oesophageal fistula, intestinal perforation, and vascular complications, as outlined in a systematic review by Tran et al. [5]. Caustic ingestions from poisons are managed with input from dedicated poison control centres.

While the hazards of certain foreign bodies, such as batteries and magnets, are well established, there is often clinical uncertainty in managing other types of ingestions for which the natural history and case progression are less clearly defined in the literature. This uncertainty can lead to high imaging rates, prolonged periods of observation, and unnecessary intervention. A better understanding of the potential harms from these less well-characterised foreign bodies could support more efficient and evidence-based care.

In this systematic review, we aimed to evaluate patterns of harm following foreign body ingestion, considering whether the ingestion was accidental or intentional, the type of object ingested, relevant patient factors, and the location of injury within the gastrointestinal tract. The primary outcome was a qualitative assessment of whether harm occurred, including the need for intervention. We aimed to find whether there were any features that indicated a very low risk of harm.

## Review

Methods

Inclusion and Exclusion Criteria

We performed an inclusive systematic review of published literature across the major medical literature databases. We included case studies and case series reporting foreign body ingestion that fulfilled our inclusion criteria in both paediatric and adult populations. We chose to exclude poisonings, batteries and magnets as foreign bodies as extensive literature is published with the known harms relating to these ingestions. We excluded case reports that did not have outcome measures, including interventions required and complications, as well as those not reported in English or involving animals. See Table 1 for details of our inclusion and exclusion criteria.

**Table 1:** Inclusion and exclusion criteria

Inclusion criteria	Exclusion criteria
Foreign body ingestion	Poisoning
Case study	Batteries
Adult	Magnets
Paediatric	Studies without outcome data
English language only	
Outcome measures for harm if present	

Database Search Strategies

We conducted an inclusive systematic review of published literature from the dates the databases began until June 2025. Databases searched included EMBASE, PubMed/Medline and CINAHL/Ebsco. Detailed search strategies for all three databases can be found in the Appendix.

Search terms used were: (Foreign body* OR foreign object* OR retained foreign material OR non-native object OR inserted object OR iatrogenic object OR implanted object OR foreign matter) AND (Ingest* or swallow*) AND (Harm OR adverse event* OR iatrogenic OR drug* OR adverse effect* OR death OR disability).

Our search was worldwide and restricted to the English language and human studies only. Medical Subject Headings (MeSH) terms for keywords used were included if present within databases. All searches and MeSH terms were approved by the University of Cambridge Medical Library. Detailed search strategies for all three databases can be found in the Appendix. Each paper was assessed by two separate reviewers, with a third reviewer available for any disagreements.

Screening and Review Process

All studies found in the literature search were screened by two separate screeners. The abstracts were reviewed to assess for correct methodology and ingestion of a suitable foreign body not outlined in our exclusion criteria. If outcome measures were not in the abstract, the study was still included on initial screening for a full-text screen. Any disagreements in initial screening between the two screeners had an optional third screener available if required.

Grey literature was not included within this systematic review as our focus was on peer-reviewed literature to identify primary evidence. Cases with aspiration secondary to ingestion were excluded when the resulting injury stemmed from respiratory tract involvement rather than the gastrointestinal tract, and thus fell outside the review's focus, unless gastrointestinal harm or intervention also occurred, in which case secondary respiratory complications were reported but did not affect inclusion.

Data extraction fields included title and author, country of origin, patient demographic, ingested foreign body, intentional/accidental, interventions and complications, and outcome.

Definition of Harm

For the purpose of this review, harm is defined as any physical complication or intervention resulting from the ingestion of a foreign body, including but not limited to perforation, obstruction, bleeding, ulceration, infection, or death (direct harm), as well as any physical intervention required due to the ingestion, such as surgery, endoscopy, colonoscopy, laparotomy or ICU admission (indirect harm). *Classification of Ingestion - Accidental vs Intentional *

For the purpose of this systematic review, we defined accidental and intentional ingestion as follows: Accidental ingestion was defined by any of the following: (a) explicit mention of "accidental"; (b) involvement of vulnerable individuals-such as children, cognitively impaired adults, elderly with dementia, or those with autism/Pica-without any indication of self-harm; (c) ingestion during meals or medical procedures, or due to a swallowing error; and (d) absence of self-harm, suicidal intent, or psychiatric diagnoses associated with deliberate ingestion. In cases where there was no reported history of ingestion, classification was based on clinical context and absence of features suggesting intentionality.

Intentional ingestion was identified if any of the following were present: (a) explicit terms such as "intentional", "deliberate", "suicidal", or "self-harm"; (b) documented psychiatric illness (e.g., borderline personality disorder, schizophrenia); (c) ingestion associated with hallucinations, delusions, or psychosis; (d) history of repeated ingestion; or (e) behaviours suggesting planning or manipulation, particularly in inpatient settings. This did not include homicide, as we wanted to focus on the more common self-harm cohort that presents in an emergency setting.

Results

Overview

**Figure 1:**
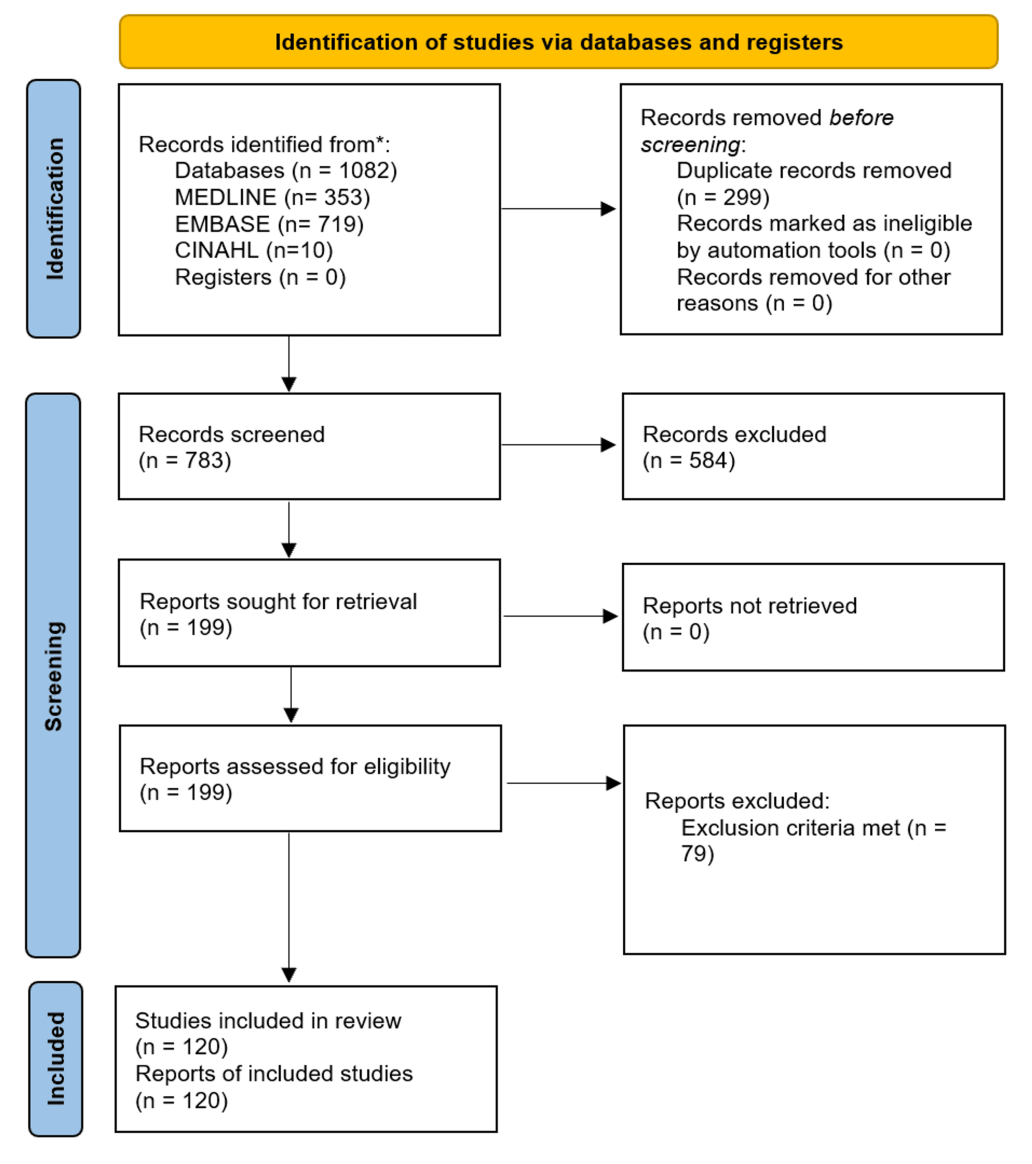
PRISMA flowchart PRISMA - Preferred Reporting Items for Systematic reviews and Meta-Analyses

Figure 1 demonstrates our Preferred Reporting Items for Systematic reviews and Meta-Analyses (PRISMA) flow diagram for our study selection process.

A systematic search of the database yielded 120 eligible studies encompassing 138 individual cases, inclusive of case series comprising multiple reports. This did not include grey literature or non-peer-reviewed journals.

Foreign bodies were categorised into types based on structural and material similarities as well as consistency across case reports. Items were grouped into clinically recognisable categories such as toothpicks, dental appliances, fish bones, chicken bones, coins, blister packs, needles, metal objects, plastics, multiple objects, and other. Fish bones and chicken bones were retained as separate categories, given their differing frequencies and clinical presentations. The 'other' category included rare or atypical items (e.g., wooden object, mussel, laryngoscope bulb, stone) that were reported too infrequently to justify a separate classification. 

Across 138 cases, a total of 147 gastrointestinal sites of harm or foreign body location were identified, reflecting that some cases involved more than one anatomical site. For descriptive analysis, we reported all sites of foreign body presence or injury; when more than one site was involved, each was recorded.

Table 2 presents a comprehensive summary of all 120 unique publications containing 138 case reports, detailing the primary outcome measures. Some publications had case series with individual case reports fitting into separate categories. Table 3 delineates comparative findings between adult and paediatric cohorts based on the location of harm of foreign bodies. Table 4 examines the relationship between foreign body ingestion, anatomical site of complication, and corresponding interventions.

**Table 2:** Summary of case reports included with outcome measurements including intent and measures of harm including mortality

Object type	Total individual cases	Accidental	Intentional	Accidental deaths	Intentional deaths	Cases with harm	Direct garm	Mortality n (%)
Toothpicks	17	17	0	3	0	17	17	3 (17.6%)
Dental	21	21	0	1	0	21	13	1 (4.8%)
Coin	8	6 (+1 child abuse)	1	1 (+ 1 child abuse)	1	8	8	3 (37.5%)
Fishbone	25	25	0	7	0	25	24	7 (28.0%)
Chicken bone	10	10	0	5	0	10	10	5 (50.0%)
Blister packs	9	9	0	2	0	9	8	2 (22.2%)
Needles	6	5	1	0	0	6	6	0 (0)
Metal objects	11	4	7	1	1	11	7	2 (18.2%)
Plastic objects	10	7	3	0	1	9	9	1 (10.0%)
Multiple objects	8	0	8	0	1	7	7	1 (12.5%)
Other objects	13	8	5	2	1	11	11	3 (23.1%)
Total	138 case reports (from 120 unique publications)	112	25	22 (+1 child abuse)	5	134	120	28 (20.3%)

**Table 3:** Comparing location of foreign bodies in adults and children

Location	Adults (n=128)	Adults %	Children (n=19)	Children %	Combined (n=147)	Combined %
Oesophagus / upper aerodigestive tract	51	39.80%	9	47.40%	60	40.80%
Stomach	20	15.60%	5	26.30%	25	17.00%
Small intestine (duodenum, jejunum, ileum)	30	23.40%	4	21.10%	34	23.10%
Large intestine (incl. appendix)	19	14.80%	0	0.00%	19	12.90%
Other (inferior vena cava, liver, passed through gastrointestinal tract, subcutaneous, unspecified bowels, mediastinum)	8	6.20%	1	5.30%	9	6.10%
Total	128	100%	19	100%	147	100%

**Table 4:** Comparing location of foreign bodies by their type

Foreign body type	Oesophagus / Upper aerodigestive tract	Stomach	Small intestine (duodenum, jejunum, ileum)	Large intestine (incl. appendix)	Other (inferior vena cava, liver, passed GI, subcutaneous, Unspecified bowels, Mediastinum)	Total
Toothpick	0	1	6	9	1	17
Dental appliance	10	2	6	2	1	21
Fish bone	14	1	5	3	2	25
Chicken bone	8	0	1	1	0	10
Coin	4	2	2	0	0	8
Blister pack	3	1	5	0	0	9
Needle	2	3	0	1	0	6
Metal object	5	4	2	1	0	12
Plastic	5	1	5	0	0	11
Multiple objects	2	4	2	1	2	11
Other	7	6	0	1	3	17
Total	60	25	34	19	9	147

Note that one paediatric case involved child abuse and was categorised separately as a non-accidental injury (NAI) rather than accidental or intentional ingestion. The case was retained in the total count but excluded from the accidental vs intentional subgroup analysis.

Table 3 compares the anatomical locations of foreign-body impaction between adults and children, highlighting variations in site distribution across the gastrointestinal tract.

Toothpick

Seventeen (17) case reports of toothpick ingestion were identified, all accidental [6-18]. The colon (9/17) was the most commonly affected site, with the sigmoid colon (6/17) most frequently involved. Perforation occurred in eleven (11/17) cases, predominantly in the sigmoid colon, but also in the upper gastrointestinal tract. Complications included fistulation to adjacent structures and abscess formation. Three (3) deaths were reported, all in males: one from caecal perforation with migration and abscess, one from fistulation with migration into the IVC, and one from cardiac tamponade following trauma, with incidental autopsy finding of diverticular perforation. Gender was mentioned in seventeen (17/17) cases: fifteen (15) male, two (2) female. Age was provided in sixteen (16/17) cases, with one (1/17) reported only as "elderly". The mean age was 65.8 years.

In the case of toothpicks, all objects passed beyond the oesophagus (17/17), with no reports of oesophageal impaction. In 12 cases, computed tomography (CT scans) were performed; a foreign body was identified in three, while in the remaining cases, CT failed to detect the toothpick, resulting in misdiagnosis and delayed definitive treatment.

The following interventions were performed (n=16/17): laparotomy (10), endoscopy-based (3), colonoscopy (2), recto-sigmoidoscopy (1), multiple procedures (2: endoscopy, colonoscopy, laparotomy 1; recto-sigmoidoscopy, angioplasty, Laparotomy 1). One case (1/17) did not report intervention.

Among 12 reported cases of toothpick ingestion with available CT imaging, the foreign body was visualised in three (25%) and not identified in nine (75%). Most scans demonstrated secondary findings, including localised abscesses, pericolic or intra-abdominal inflammation, and contained perforations, often misinterpreted as diverticulitis or inflammatory masses. Only a few scans revealed a faint linear or radiopaque structure suggestive of a wooden toothpick. Overall, CT showed substantially lower sensitivity for wooden or radiolucent objects, with detection usually limited to associated complications rather than the foreign body itself.

Table 5 demonstrates case reports of toothpick ingestion in more detail.

**Table 5:** Case reports of toothpick ingestion (17 reported cases from 13 publications) y - year; M - male; F - female; CT - computed tomography; OGD - oesophago-gastro-duodenoscopy; IVC - inferior vena cava

Author	Country	Age / sex	Object	Location	Intent	Interventions/complications	Outcome
Ricci et al. (2013) [6]	Italy	80y F	Toothpick	Sigmoid colon	Accidental	Two colonoscopies; sigmoid perforation	Survived
Ioannidis et al. (2010) [7]	Greece	59y M	Toothpick	Ileum	Accidental	CT missed it; misdiagnosed as Crohn’s; treated with steroids; exploratory laparotomy revealed fistula	Survived
Wilcher et al. (2010) [8]	Australia	Elderly M	Toothpick	Sigmoid colon	Accidental	Perforated diverticulum found on autopsy; death due to cardiac tamponade from a car crash	Death (unrelated)
Chung et al. (2008) [9]	Korea	52y M	Toothpick	Sigmoid colon	Accidental	Sigmoid impaction; pseudodiverticulum; removed via colonoscopy	Survived
Hauser et al. (1994)[10]	Austria	74y M	Toothpick	Caecum	Accidental	Septic shock; psoas abscess on CT; caecal perforation; retroperitoneal migration; toothpick found in abscess	Death
Martyn et al. (2023)[11]	US	57y M	Toothpick	Duodenum	Accidental	CT missed object; removed by OGD with no complications	Survived
Evola et al. (2023) [12]	Italy	72y M	Toothpick	Sigmoid colon	Accidental	CT showed pericolic abscess; sigmoid perforation; laparotomy; colorectal anastomosis; partial omentectomy; protective ileostomy	Survived
Mahmoud et al. (2023) [13]	US	79y F	Toothpick	Stomach	Accidental	CT missed object; stomach perforation with contained leak; ICU after intubation	Survived
Sennett et al. (2022) [14]	US	72y M	Toothpick	Caecum	Accidental	CT showed a hyperdense object perforating the caecum; right hemicolectomy with primary anastomosis	Survived
Valiyeva et al. (2020) [15]	Italy	77y M	Toothpick	Sigmoid colon	Accidental	Misdiagnosed as diverticulitis; later CT showed aneurysm; sigmoidoscopy revealed a toothpick; entero-iliac fistula; stent placed	Survived
James et al. (2020) [16]	US	79y M	Toothpick	Duodenum, Liver	Accidental	Hepatic abscess; CT found foreign body; multiple endoscopic attempts due to breakage; stenting and drainage	Survived
Reginelli et al. (2016) [17]	Italy	61y M	Toothpick	Jejunum	Accidental	CT found a foreign body; exploratory laparotomy showed jejunal perforation	Survived
Cockerill et al. (1983) [18]	US	66y M	Toothpick	Proximal ileum	Accidental	Presumed appendicitis; laparotomy: appendix normal; ileum adherent to bowel; toothpick tip protruding; perforation closed	Survived
	US	73y M	Toothpick	Ileum	Accidental	Chronic epigastric pain; laparotomy showed adhesions and a mass; toothpick perforating the small bowel; resected	Survived
	US	51y M	Toothpick	IVC	Accidental	Fever, chills; prior negative laparotomy; polymicrobial sepsis; autopsy: toothpick in IVC thrombus	Death
	US	64y M	Toothpick	Sigmoid colon	Accidental	Chronic right hip osteomyelitis; gut flora; CT and Barium enema: sigmoid fistula to hip abscess; laparotomy: toothpick removed	Survived
	US	37y M	Toothpick	Posterior rectum	Accidental	Ate omelette with toothpicks; episodic pain; eosinophilia; laparotomy: toothpick in posterior rectum	Survived

Dental

Twenty-one (21) case reports of dental appliance ingestion were identified [19-38]. Nine (9/21) objects were removed from the oesophagus, one (1/21) from the hypopharynx, two (2/21) from the stomach, seven (7/21) from the small intestines, and one (1/21) from the sigmoid colon. Two (2/21) objects-an endodontic file and a dental bur-passed spontaneously without intervention. One (1/21) death occurred in a male patient. Demographics were reported in all cases: 14 (14/21) male and seven (7/21) female. The average age was 44.1 years. The following interventions were performed (n=19/21): 13 (13/21) endoscopies, three (3/21) laparotomies, one (1/21) colonoscopy, and one (1/21) gastroscopy with duodenal Kocherisation. Two (2/21) cases did not report interventions.

Table 6 demonstrates dental case reports in detail.

**Table 6:** Dental ingestion case reports (21 reported cases from 20 publications) y - year; M - male; F - female; CT - computed tomography; OGD - oesophago-gastro-duodenoscopy

Author(s)	Country	Age/ sex	Object	Location	Intent	Intervention/ complications	Outcome
Kim et al. (2021) [19]	UK	62y M	Dentures	Oesophagus	Accidental	Intraoral forceps	Survived
Rohida et al. (2011) [20]	US	12y M	Twin-block fragment	Oesophagus	Accidental	Endoscopy	Survived
Rana et al. (2009) [21]	UK	82 y M	Dental plate	Hypopharynx	Accidental	Endoscopy and biopsy (masqueraded as malignancy)	Survived
Kuo SC et al (2008) [22]	Taiwan	51 y M	Endodontic file	Passed through the GI tract	Accidental	Observation	Survived
Ekanem et al. (2005) [23]	Nigeria	56 y M	Acrylic denture	Oesophagus	Accidental	Oesophageal laceration and severe haemorrhage	Death
Huang et al. (2024)[24]	China	31y F	Fractured orthodontic aligner	Oesophagus	Accidental	Endoscopic extraction	Survived
Jesani et al. (2024) [25]	UK	60s M	Denture	Oesophagus	Accidental	Trans-oesophageal fistula, OGD, oesophagoscopy, thoracotomy, tracheostomy, pharyngolaryngectomy, feeding jejunostomy, eight-month recovery	Survived with morbidity
Sghaier et al. (2023) [26]	Tunisia	85y F	Dentures	Terminal Ileum	Accidental	Acute intestinal occlusion, laparotomy	Survived
Golubykh et al. (2022) [27]	US	53y F	Dental drill	Oesophagus	Accidental	Intubation, OGD removal	Survived
Incitti et al. (2021) [28]	US	52y M	Dental bur	Appendix	Accidental	CT: metallic foreign body in the proximal appendix, colonoscopic removal	Survived
Flanagan et al. (2018) [29]	Ireland	67y F	Dental plate	Sigmoid colon	Accidental	Erosion through the colon wall, sigmoid colectomy, colo-colic anastomosis	Survived
Shor et al. (2015) [30]	US	43y F	Tooth	Oesophagus	Accidental	Endoscopic retrieval using Roth net	Survived
Gachabayov et al. (2015) [31]	Russia	54y F	Retractable one-tooth denture	Small intestine	Accidental	Small bowel obstruction, laparotomy, enterotomy with retrieval	Survived
	Russia	31y M	Fixed one-tooth prosthesis	Ileocaecal valve	Accidental	OGD, partial obstruction, spontaneously passed	Survived
Jain et al. (2015) [32]	India	26y M	23G hypodermic needle	Stomach	Accidental	Embedded in the gastric wall, removed via flexible endoscopy after an inconclusive rigid oesophagoscopy	Survived
Tiller et al. (2014) [33]	Germany	37y F	Kobayashi ligature	Duodenum → pancreas migration	Accidental	Chronic pancreatitis, removed endoscopically via snare under ultrasound guidance	Survived
Yilmaz et al. (2012) [34]	Turkey	33y M	Lower dental prosthesis	Duodenum	Accidental	Gastrotomy and duodenal Kocherization	Survived
Soulsby et al. (2015) [35]	UK	46y M	Partial denture	Oesophagus	Accidental	Full-thickness mucosal perforation, oesophagoscopy, antibiotics	Survived
Haug et al. (1993) [36]	USA	28y M	Dental fixation wire	Stomach	Accidental	Endoscopic retrieval of a wire from the proximal stomach	Survived
Amarlal et al. (2009) [37]	India	4y M	Dental bur	Duodenum at time of imaging – passed through the GI tract	Accidental	Observation, passed in stool within 24 hours with no complications	Survived
Hinkle (1987) [38]	US	14y M	retainer / mandibular anterior realignment appliance	Oesophagus	accidental	Imaging showed a retainer lodged mid-oesophagus near the trachea. Retrieved using an oesophagoscope, forceps.	Survived

Chicken Bone

Ten (10) case reports of chicken bone ingestion were identified [39-48]. The bone was lodged in the oesophagus in seven (7/10) cases, and in the oesophagus and stomach in one (1/10). Complications were frequent and included perforation of the oesophagus (5/10), right common carotid artery (1/10), and ileum (1/10). Reported fistulations involved the aorta-oesophageal (1/10), colo-vesical (1/10), and oesophageal-arterial (1/10) tracts. All vascular complications led to mortality. Five (5/10) patients died from complications related to perforation and/or migration. Demographic details were available in nine (9/10) cases: three (3/9) male and six (6/9) female. The mean age was 60.7 years. Deaths occurred in three (3/6) females and two (2/3) males. The following interventions were performed (n=9/10): three (3/10) laparotomies, one (1/10) colonoscopy, three (3/10) endoscopies, one (1/10) endoscopy with vascular intervention and ENT drainage, and one (1/10) cervical incision. One (1/10) case did not report intervention and resulted in death.

Table 7 demonstrates case reports of chicken bone ingestion.

**Table 7:** Case reports of chicken bone ingestion (10 reported cases from 10 publications) y - year; M - male; F - female; CT - computed tomography; OGD - oesophago-gastro-duodenoscopy

Author(s)	Country	Age/ sex	Object	Location	Intent	Interventions/ complications	Outcome
Clements et al. (2013) [39]	US	Not stated	Chicken bone	Bladder and colon	Accidental	Colovesical fistula. Partial removal under colonoscopy; the remaining fragment passed spontaneously per urethra.	Survived
Peonim et al. (2010) [40]	Thailand	42y F	Chicken bone	Oesophagus and stomach	Accidental	Oesophageal and gastric bleeding, oesophageal perforation, arterial-oesophageal fistula. Laparotomy. Arrested, death from hypovolaemic shock.	Death
Simic et al. (1998) [41]	US	64y M	Chicken bone	Oesophagus	Accidental	Haematemesis, laparotomy. Death. Post-mortem: oesophageal perforation and decubitus ulcer with small vessel erosion.	Death
Russo et al. (1986) [42]	US	81y F	Chicken bone	Oesophagus	Accidental	Oesophageal and right common carotid artery perforation, resulting in rapid exsanguination.	Death
Dev et al. (2023) [43]	Nepal	54y M	Chicken bone	Ileum	Accidental	Ileal perforation. Managed with laparotomy.	Survived
Musa et al. (2021) [44]	US	55y F	Chicken bone	Oesophagus	Accidental	CT: hyperdense foreign body in the antrum, Impacted prepyloric chicken bone removed endoscopically.	Survived
Sabuncuoglu et al. (2015) [45]	Turkey	54y F	Chicken bone	Oesophagus	Accidental	Oesophageal perforation. Neck surgery: transverse cervical incision, transoral extraction, oesophageal repair with 3/0 vicryl.	Survived
Sonzogni et al. (1996) [46]	Italy	48y F	Chicken bone	Oesophagus	Accidental	Sudden haematemesis, Oesophagodynia, dysphagia. Recurrent haematemesis (3000 mL) and melaena. OGD: clot 0.5 cm on oesophageal wall. Post-mortem: 1 cm aorto-oesophageal fistula.	Death
Doi (2010) [47]	US	90y F	Chicken bone	Oesophagus	Accidental	Sepsis, oesophageal perforation, septic IJV thrombophlebitis and abscess. OGD removed chicken bone from the cervical oesophagus, drained the neck abscess. Vascular surgery resected a thrombosed, fibrotic, and purulent internal jugular vein segment.	Survived, discharged to hospice
Belanny et al. (2022) [48]	Indonesia / Armenia	58y M	Chicken meat with bone	Oesophagus	Accidental	Chicken meat in the oesophagus removed by oesophagoscopy. Seven days later: severe dysphagia, neck emphysema. Imaging: retropharyngeal/parapharyngeal emphysema, suspected oesophageal perforation with gas-forming infection. Deteriorated and died.	Death

Fish Bone

Twenty-five (25) case reports of fish bone ingestion were identified [48-69]. The most common location was the oesophagus (12/25). Other reported sites included the small intestine (3/25, of which 2/25 were ileum), large intestine (3/25: caecum 1/25, ascending colon 1/25, transverse colon 1/25), liver (1/25), stomach (1/25), subcutaneous tissue (1/25), paratracheal region (1/25), and submandibular region (1/25). Perforation occurred in 12 (12/25) cases, most frequently in the oesophagus (7/12). Other perforation sites included the ileum (2/12), small intestine unspecified (1/12), and large intestine (2/12: caecum 1/12, ascending colon 1/12). Seven (7/25) deaths were reported: four (4/13) females and three (3/12) males. Causes included severe infections/sepsis (mediastinitis, septic shock, pneumonia complications), perforation-related complications, and haemorrhagic shock in one (1/25) gastric case. Demographic data were available in all cases: 12 (12/25) males and 13 (13/25) females. The mean age was 56.8 years.

The following interventions were performed (n=25): Endoscopy-based (11: alone 2, with endovascular repair 2, with thoracotomy + intubation 1, with laryngoscopy + thoracotomy + transoral incision 1, with intubation 1, with intensive treatment unit (ITU) 1, with surgical drainage 1, with thoracotomy 1, with laryngoscopy 1), laparoscopy (2), laparotomy (4, including 1 with ITU), thyrotomy (1), Incision/drainage procedures (3), laminectomy + corpectomy + spinal fusion (1). Three cases did not report interventions.

Among 25 reported cases of fish bone ingestion, CT was performed in 16 (64%). The foreign body was visualised in 12 cases (75%) and not seen in four (25%). In most positive cases, CT demonstrated a linear or needle-shaped radiopaque structure consistent with a calcified fish bone, frequently penetrating the oesophageal wall, thyroid gland, mediastinum, or gastrointestinal tract. Common associated findings included localised perforation, abscess, soft-tissue inflammation, and, in some instances, vascular complications such as pseudoaneurysm or aorto-oesophageal fistula. In the minority of negative scans, CT showed only indirect features such as wall thickening, fat stranding, air collections, or localised fluid without clear visualisation of the foreign body.

Table 8 shows case reports of fish bone ingestion.

**Table 8:** Case reports of fish bone ingestion (25 reported cases from 22 publications) y - year; M - male; F - female; CT - computed tomography; OGD - oesophago-gastro-duodenoscopy; ITU - intensive care unit; PCI - percutaneous coronary intervention; AKI - acute kidney injury; MODS - multiple organ dysfunction syndrome; RV - right ventricular; MI - myocardial infarction; EUS - endoscopic ultrasound

Author(s)	Country	Age/ sex	Object	Location	Intent	Interventions/ complications	Outcome
Belanny et al. (2022) [48]	Indonesia	39y F	Fish bone	Oesophagus	Accidental	Fish bone retropharyngeal abscess. Foreign body at C5. Surgical drainage, OGD done, no foreign body found. Treated with ceftriaxone, metronidazole. Culture: Streptococcus constellatus. Post-op day 6 CT: fluid, gas at C6–C7 retropharynx, no foreign body. Antibiotic switched to levofloxacin, daily drainage continued. Chest X-ray: bronchopneumonia. ICU stay 19 days, died from pneumonia complications.	Death
	Indonesia	73y F	Fish bone	Oesophagus	Accidental	Fibre optic laryngoscopy showed bulging retropharynx, pooled secretions in vallecula and piriform sinuses, and no foreign body. X-ray showed C1–C4 soft tissue lucency, widened retropharyngeal space, and no radio-opaque object. CT not performed due to renal impairment. Diagnosed with retropharyngeal abscess and pre-renal AKI. Surgical drainage was performed. Culture grew *Streptococcus anginosus*. Postoperatively, ICU stay for four days with metabolic acidosis, septic shock, electrolyte imbalance, hypoalbuminemia, and MODS.	Death
Zhou et al. (2023) [49]	China	66y M	Fish bone	Oesophagus	Accidental	CT confirmed oesophageal perforation into the aortic arch, thoracic endovascular aortic repair, and fish bone was removed via endoscopy	Survived
Li et al. (2019) [50]	China	58y M	Fishbone	Liver	Accidental	Laparoscopic left hepatectomy for CT-confirmed liver abscess	Survived
Chen et al. (2011) [51]	Taiwan	50y F	Fish bone	Oesophagus	Accidental	Intra-thyroid abscess with fish bone penetrating from the cervical oesophagus into the thyroid, thyrotomy	Survived
Blanco Ramos et al. (2009) [52]	Spain	59y F	Fish bone	Oesophagus	Accidental	Mediastinitis, endoscopy, intubation, thoracotomy, necrotic ulcers in oesophagus; had seizures, cardiac arrest, acute MI, livedo reticularis, facial angioedema, severe RV dysfunction, oesophageal fistula	Survived
Rodriguez-Hermosa et al. (2009) [53]	Spain	58y F	Fish bone	Small intestine	Accidental	Small bowel perforation, emergency surgery - segmental intestinal resection, intensive care admission	Death
Nozoe et al. (1998) [54]	Japan	74y M	Fish bone	Oesophagus	Accidental	Oesophageal perforation, conservative management	Survived
Sannohe et al. (1998) [55]	Japan	58y F	Fish bone	Stomach	Accidental	Autopsy finding of gastric haemorrhage, died from haemorrhagic shock	Death
Joseph et al. (2025) [56]	US	49y F	Fish bone	Oesophagus	Accidental	Abscess requiring intubation, endoscopic submucosal dissection and fish bone extraction	Survived
Husain et al. (2022) [57]	Saudi Arabia	71y F	Fish bone	Transverse colon	Accidental	CT: Transverse colon thickening, concerns for concealed perforation, conservative management	Survived
Munasinghe et al. (2022) [58]	Sri Lanka	61y M	Fish bone	Ileum	Accidental	Acute faecal peritonitis secondary to ileal perforation	Death
Yamada et al. (2022) [59]	Japan	77y F	Fish bone	Oesophagus	Accidental	Cervical oesophageal perforation, pneumomediastinum, OGD and removal of fish bone with snare forceps	Survived
Koh et al. (2021)[60]	Malaysia	40y F	Fish bone	Subcutaneous tissue	Accidental	Fish bone migrating to subcutaneous neck tissue, incision and removal under local anaesthesia	Survived
	Malaysia	29y F	Fish bone	Oesophagus	Accidental	Posterior oesophageal wall perforation with C3-T2 prevertebral abscess, transoral incision and drainage via direct laryngoscopy, thoracic empyema, thoracotomy, OGD.	Survived
	Malaysia	47y M	Fish bone	Paratracheal (from oesophageal perforation)	Accidental	Respiratory distress, subcutaneous crepitus, intubation, hollow viscus perforation, OGD, ITU due to septicaemia, MODS	Death on day 2 of admission
Dung et al. (2021) [61]	Vietnam	51y M	Fish bone	Caecum	Accidental	Caecal perforation, laparoscopic cecectomy	Survived
Basaranoglu (2019) [62]	Turkey	63y M	Fish bone	Small intestine	Accidental	OGD, EUS, suppurative gastritis, liver abscess	Survived
Strohaker et al. (2018) [63]	Germany	56y M	Fish bone	Ileum	Accidental	CT: Intestinal perforation involving appendix, laparoscopic surgery converted to laparotomy due to adhesions, appendectomy	Survived
Yamamoto et al. (2015) [64]	Japan	69y M	Fish bone	Ascending colon	Accidental	CT: hyperdense foreign body in abdomen, Intra-abdominal abscess due to ascending colon perforation, surgical drainage via flank incision	Survived
Sia et al. (2013) [65]	Malaysia	54y M	Fish bone	Oesophagus	Accidental	Hematemesis, OGD, oesophageal perforation, proximal descending aortic pseudoaneurysm, aorto-oesophageal fistula, thoracic endovascular aortic repair	Death from mediastinal sepsis
Chen et al. (2010) [66]	Taiwan	58y M	Fish bone	Submandibular region	Accidental	Ludwig's angina, incision and drainage	Survived
Friedlander et al. (2013) [67]	US	59y F	Fish bone	Oesophagus	Accidental	Odynophagia, haemoptysis; imaging showed upper thoracic cavitary lesion. Developed acute paraplegia while awaiting endoscopy. Imaging revealed T2-T5 epidural abscess with T4–T5 diskitis and osteomyelitis. Underwent laminectomy T2–T10, then T3–T4 corpectomies and T1–T10 posterior spinal fusion.	Survived with morbidity L4 AIS D
Patel et al. (2023) [68]	New Zealand	33y M	Fish bone	Oesophagus	Accidental	CT showed 2.2 × 1.5 × 1.5 cm pseudoaneurysm from aortic arch. Emergency repair via left thoracotomy with bypass, deep hypothermic arrest, aortic graft. Aortitis noted; 1 cm aortic hole feeding hematoma cavity. Gastroscopy post-op identified and closed oesophageal perforation. Cultures grew Streptococcus intermedius and Candida albicans. Reoperation on day 3 for washout. Antimicrobials, NG feeding until resolution. Discharged day 33. Completed 12-month antifungals. CT at five years was normal.	Survived
Zhao et al. (2019) [69]	China	68y F	Fish bone	Ileum	Accidental	Three days post-PCI developed acute right-lower-quadrant pain; CT showed fish bone penetrating ileum with local leakage and pus. Emergency laparotomy with partial small bowel resection performed.	Survived

Coins

Eight (8) case reports of coin ingestion were identified [70-77]. Four (4/8) involved paediatric patients (ages 4.5 months to three years). In all paediatric cases, the coin became lodged in the oesophagus (4/4). Two (2/4) paediatric patients died: one from oesophago-aortic fistula with aortic perforation, and one from gastric distension with possible aspiration (complicated by child abuse). The remaining two (2/4) paediatric cases developed oesophageal perforation with migration of the coin into the mediastinum (1/4) and the T1 cavity anterior to the oesophagus (1/4); both were successfully managed with thoracoscopic removal. Four (4/8) cases occurred in adults. In adults, coin locations differed: stomach (2/4) and ileum (2/4). Adult complications included small intestine perforation (1/4), small bowel obstruction (1/4), pyloric ulceration (1/4), and acute copper toxicity following ingestion of a large number of coins (275 copper coins) (1/4). Demographic data were available for all cases: four (4/8) male and four (4/8) female. The mean age was 35.1 years.

The following interventions were performed (n=5/8): Endoscopy-based (4: alone 1, with thoracoscopy 1, with bronchoscopy & thoracoscopy 1, with pouchoscopy 1), laparotomy (1). In three (3/8) cases, no intervention was reported, and all three died.

Table 9 demonstrates case reports for coin ingestion.

**Table 9:** Case reports for coin ingestion (eight reported cases from eight publications) y - year; M - male; F - female; CT - computed tomography

Author(s)	Country	Age/ sex	Object(s)	Location	Intent	Interventions/ complications	Outcome
Dahiya et al. (1999) [70]	US	3y M	Coin	Oesophagus	Accidental	Coin eroded through the oesophagus into the aorta, forming an oesophagoaortic fistula.	Death – from aortic perforation
Nolte et al. (1993) [71]	US	4.5 month F	3 coins	Oesophagus	Child abuse	Autopsy revealed three coins in the oesophagus, multiple bruises, acute and healing limb fractures, old aspirated material in lungs, and pulmonary fat emboli	Death possibly contributed to by fat emboli and complications from oesophageal foreign bodies.
Yelin et al. (1987) [72]	US	58y F	275 copper coins	Stomach	Intentional	Acute toxic phase of chronic copper poisoning following massive coin ingestion, leading to haemolytic anaemia, hepatic failure, and multiorgan dysfunction.	Death – acute copper toxicity
Saeed et al. (2020) [73]	US	76y M	2 quarter coins	Terminal Ileum	Accidental	Obstruction and small perforation. Required exploratory laparotomy with right hemicolectomy and anastomosis.	Survived
Ratra et al. (2017) [74]	US	72y M	Penny coin	Distal Ileum	Accidental	CT: Small bowel stricture and obstruction. Managed with pouchoscopy.	Survived
Colizzo et al. (2016) [75]	US	68y M	Dime and quarter	Stomach	Accidental	Pyloric ulcer. Coins removed with endoscopy and Roth net. Treated with proton pump inhibitors.	Survived
Dumeer et al. (2013) [76]	US	15 mo F	2 coins	Oesophagus and mediastinum	Accidental	One coin removed via endoscopy. Second coin migrated to mediastinum, removed with thoracoscopy. Developed oesophageal stricture requiring dilation.	Survived
Raval et al. (2004) [77]	US	23 mo F	Penny coin	Oesophagus	Accidental	Long-standing dysphagia. Coin not seen on endoscopy. CT showed tracheal compression. Thoracoscopic removal of coin from T1 cavity anterior to oesophagus performed. Discharged after 3 days.	Survived

Blister Backs (Press-Through Packages)

Nine (9) case reports of blister pack ingestion were identified, all accidental [78-84]. The most common site was the small intestine (5/9). All five (5/5) small intestine cases resulted in perforation, with one (1/5) leading to death. The oesophagus was the second most common location (3/9). Within the oesophagus, one (1/3) case developed a tracheo-oesophageal fistula, one (1/3) case resulted in oesophageal perforation and death, and one (1/3) case was successfully managed with endoscopic removal (OGD). One (1/9) case passed through the gastrointestinal tract without complications. Demographic data were available for all cases: six (6/9) male and three (3/9) female. The mean age was 74 years, with eight (8/9) patients aged >70 years. Two (2/9) deaths occurred, both in males.

The following interventions were performed (n=7/9): laparotomy (4), endoscopy-based (3: OGD removal 1, unspecified endoscopy 2). In two (2/9) cases, no intervention was reported (both fatal).

Table 10 lists case reports for blister pack ingestion.

**Table 10:** Case reports for blister pack (press through package) ingestion (nine reported cases from seven publications) y - year; M - male; F - female; CT - computed tomograph

Author(s)	Country	Age/ sex	Object	Location	Intent	Interventions/ complications	Outcome
Simo Alari et al. (2018) [78]	France	72y F	Blister pack	Ileum	Accidental	Mesenteric inflammation, four contained perforations in ileum, 30 cm bowel resection	Survived
Hovde et al. (2013) [79]	Norway	72y M	Blister pack	Oesophagus	Accidental	Oesophageo-tracheal fistula with stenosis and oesophageal ulceration, OGD with oesophageal stenting and removal on follow-up	Survived
Bosmans et al. (2006) [80]	Belgium	75y F	Blister pack	Terminal Ileum	Accidental	CT: blister pack in terminal ileum. Small bowel perforation, laparotomy and enterotomy performed.	Survived
Hou et al. (2006) [81]	Taiwan	76y M	Blister pack	Oesophagus	Accidental	Radiopaque nodular density over prevertebral region C6–C7, emergent upper GI endoscopy showed PTP with pill lodged 20 cm from incisor, removed with forceps	Survived
	Taiwan	56y F	Blister pack	Stomach, then passed through GI tract	Accidental	Plain abdominal film showed radiopaque nodular density, 0.8 × 0.4 cm, in epigastric region. Emergency endoscopy inconclusive. Passed spontaneously	Survived
	Taiwan	78y M	Blister pack	Ileum	Accidental	CT: staple-like foreign body. Arrest in passage, local peritonitis. Laparotomy: perforation and abscess. Segmental resection performed	Survived
Chiu et al. (2007) [82]	Taiwan	78y M	Blister pack	Ileum	Accidental	CT: Distal ileal perforation and abscess formation, adhesions, laparotomy	Survived
Yu et al. (2022) [83]	South Korea	79y M	Blister pack	Oesophagus	Accidental	Refused radiological tests, Oesophageal perforation	Death
Hashizume et al. (2015) [84]	Japan	80y M	Blister pack	Small intestine	Accidental	CT showed only bowel wall oedema, treated with antibiotics. Autopsy revealed small bowel perforation	Death

Needles

Six (6) case reports of needle ingestion were identified [85-90]. Three (3/6) needles were located in the stomach at the time of intervention. Five (5/6) cases were accidental, and one (1/6) was intentional. All six (6/6) patients survived. Complications were varied without a clear dominant pattern. Demographic data were available for all cases: four (4/6) female and two (2/6) male. The mean age was 23.6 years.

The following interventions were performed (n=6/6): endoscopy-based (2), laparoscopy (1), laparotomy (1), median sternotomy (1), incision and drainage (1).

Table 11 lists case reports for needle ingestion.

**Table 11:** Reported cases of needle ingestion (six reported cases from six publications) y - year; M - male; F - female; CT - computed tomograph; IJV - internal jugular vein

Author(s)	Country	Age/ sex	Object	Location	Intent	Interventions/ complications	Outcome
Yolcu et al. (2014) [85]	Turkey	35y M	Sewing needle	Oesophagus and Pericardium	Accidental	Pericardial effusion; median sternotomy; tip found in oesophagus and pericardium	Survived
Kocourkova et al. (2009) [86]	Czech Republic	16y F	Multiple sewing needles	Stomach	Intentional	Intentional staggered self-harm. Laparotomy performed; 11 needles extracted without complications	Survived
Stojkovic et al. (2023) [87]	Serbia	45y F	Sewing pin	Stomach	Accidental	Sewing pin stuck in pylorus; extracted via endoscopy	Survived
Krane et al. (2022) [88]	US	83y M	Needle	Stomach	Accidental	Presented with melena; antral ulcerations penetrating the stomach mucosa; managed via OGD	Survived
Tustumi et al. (2020) [89]	Brazil	64y F	Needle	Appendix	Accidental	Perforated appendicitis due to needle; laparoscopic appendicectomy performed	Survived
Bendiouri et al. (2021) [90]	Morocco	40y F	Sewing needle	Pharynx	Accidental	CT: Parapharyngeal cellulitis, left IJV–subclavian thrombosis, metallic foreign body; surgery revealed 4 cm sewing needle through right IJV, sternocleidomastoid, and thyroid cartilage. Needle removed, vein ligated, abscess drained, and tissue debrided. Discharged on day seven.	Survived

Metal Objects

Eleven (11) case reports of metal object ingestion were identified [48,91-100]. The most frequent location was the oesophagus (6/11), followed by the stomach (2/11), small intestine, including the ileum (2/11), and one (1/11) case where the object was identified in both the caecum and stomach. Four (4/11) cases were accidental, mostly involving small metal objects, while seven (7/11) were intentional ingestions. Two (2/11) fatalities occurred: one (1/11) accidental case due to oesophageal bleeding and one (1/11) intentional case due to septic shock secondary to small intestinal obstruction. Demographic data were available in all cases: six (6/11) male and five (5/11) female. One (1/6) male and one (1/5) female died. Mean age was 26.3 years (range: 12-46 years).

The following interventions were performed (n=11/11): endoscopy-based (5: alone 2, with laparoscopy 1, with surgery + C-arm 1, with intubation + laryngoscopy 1), laparotomy-based (2: laparotomy + gastrotomy 1, laparotomy 1), gastrotomy + colotomy (1), endoscopy + transcervical oesophagotomy + thoracic oesophageal surgery (1), colonoscopy (1), tracheostomy + laryngoscopy (1).

Table 12 lists case reports for ingestion of metallic objects.

**Table 12:** Reported cases of ingestion of metal objects (11 reported cases from 11 publications) y - year; M - male; F - female; CT - computed tomograph

Author	Country	Age/ sex	Object	Location	Intent	Interventions/ complications	Outcome
Mesfin et al. (2022) [91]	Ethiopia	22y M	Metallic nails, wires and needles	Stomach	Intentional	Gastric outlet obstruction, exploratory laparotomy and gastrotomy under general anaesthesia; 60 curved and straight nails, needles and wires removed	Survived, discharged POD 4
Belanny et al. (2022) [48]	Indonesia / Armenia	13y M	Iron wire	Oesophagus	Accidental	CT showed bone-density foreign body ±2 cm penetrating posterior oesophageal wall forming retropharyngeal abscess. Initial surgery: abscess drainage, transoral esophagoscopy, foreign body not found. Empirical antibiotics given. Repeat CT: foreign body still present, new left parapharyngeal abscess. Second oesophagoscopy: no findings. Third surgery with C-arm: iron wire ±2.2 cm removed posterior to Oesophagus. Massive neck bleeding 3 days later; carotid ligation, Post-op coma, progressive deterioration, death 6 days later from suspected MODS due to haemorrhagic shock.	Death
Santos et al. (2022) [92]	US	18y M	Wire brush fragment	Ileum	Accidental	Bleeding PR, Bowel obstruction, Colonoscopy: metallic object was seen protruding near the ileocecal valve, CT: several nonspecific enlarged fluid-filled loops of small bowel within the right lower quadrant particularly at the terminal ileum	Survived, symptom free 4 days later
Guarner et al. (2018) [93]	UK	24Y F	Table knife	GEJ	Intentional	Chest X-ray: foreign body at gastro-oesophageal junction, no pneumoperitoneum, no pneumomediastinum. Failed endoscopy; laparoscopic approach chosen. Gastrotomy on anterior gastric body, knife removed via 12 mm trocar wound left flank.	Survived
Hayes et al. (2024) [94]	US	43Y F	Six knives	Small intestine	Intentional	Laporotomy to relieve obstruction in small intestine, extensive fibrosis seen from previous perforations relating to ingestion, septic shock	Death
Herrera-Zamora et al. (2016) [95]	US	46y M	Crucifix foreign body	Oesophagus	Accidental in context of drug addiction	Chest X-ray: radiopaque object mid-Oesophagus. Endoscopy: metallic foreign body 20 cm below dental arch, embedded, not removable. CT: T-shaped object anterior to T1–T3. Transcervical Oesophagotomy with thoracic Oesophageal myotomy for foreign body removal. Discharged with good oral intake.	Survived
Delgado Salazar et al. (2020) [96]	Eucador	31y F	Two razor blades	Stomach and caecum	Intentional	Upper endoscopy abandoned due to risk of perforation as razor blade embedded in mucosa, CT and X‑ray localization, followed by gastrostomy and colotomy for removal of both blades	Survived
Tsesmeli et al. (2007) [97]	Greece	16y F	Tongue-ring clip	Stomach	Accidental	Foreign bodies in stomach and gut; upper GI endoscopy with removal using biopsy forceps	Survived, discharged same day
Wright et al. (2021) [98]	US	29y M	Crucifix necklace	Oesophagus	Intentional	Intubation for airway protection; initial EGD extraction unsuccessful; crucifix removed via direct laryngoscopy using Lindholm laryngoscope	Survived, transferred to psychiatric hospital
Lai et al. (2022) [99]	US	32y F	Toilet paper holder bracket	upper aerodigestive tract / upper oesophagus	Intentional	Failed nasotracheal and orotracheal attempts at fibreoptic intubation, emergent tracheostomy, removal attempts with direct laryngoscopy, followed by Dedo laryngoscope.	Survived
Al‑Faham et al. (2020) [100]	Iraq	42y M	Spanner	Oesophagus	Intentional	Completely obstructing the oesophagus. Removed via rigid oesophagoscopy under anaesthesia	Survived

Plastic Objects

Ten (10) case reports of plastic ingestion were identified, involving five (5/10) adults and five (5/10) children [50, 101-108]. The most common anatomical location was the small intestine (5/10: duodenum 1/10, jejunum 1/10, ileum 1/10, duodenum + liver 1/10, unspecified small bowel 1/10). Other sites included the oesophagus (3/10) and the oesophagus-gastroesophageal junction-stomach (1/10). Three (3/10) cases were intentional ingestions. One (1/10) intentional ingestion resulted in death secondary to a pyogenic abscess. Demographic data were reported in all cases: six (6/10) male and four (4/10) female. Age was provided in nine (9/10) cases, with a mean age of 25.5 years.

The following interventions were performed (n=9/10): endoscopy-based (3: alone 1, with laryngoscopy 2), laparotomy-based (3: laparotomy 2, laparotomy + tracheostomy 1), intraoral surgical removal + Intubation (1), surgical removal + tracheostomy (1). In one (1/10) case, no intervention was performed.

Table 13 lists case reports for plastic object ingestion.

**Table 13:** Reported cases of plastic object ingestion (10 reported cases from nine publications) y - year; M - male; F - female; CT - computed tomograph; OGD - oesophago-gastro-duodenoscopy

Author(s)	Country	Age/ sex	Object(s)	Location	Intent	Interventions/ complications	Outcome
Zhao et al. (2022) [50]	China	Male adult prisoner	Toothbrush handle	Duodenum and liver	Intentional	Toothbrush-induced hepatic abscess, 14.5-cm-long toothbrush handle was found in the duodenum and had penetrated the right lobe of the liver, ingested 16m prior	Died from pyogenic hepatic abscess diagnosed at autopsy
Pont et al. (2021) [101]	US	38y M	Toothbrush	Oesophagus, GE junction, stomach	Intentional	XR unrevealing, non-contrast CT: long foreign object in the stomach, multiple ingestions, removed with upper endoscopy under general anaesthesia	Survived
Kim et al. (2014) [102]	South Korea	44y M	Toothbrush	Pharynx	Intentional	Intra oral surgical removal under anaesthesia and Nasotracheal intubation, repair of uvular laceration, small hypopharyngeal perforation	Survived, discharged after 10 days
Suraj et al. (2024) [103]	Nepal	16y F	Head portion of doll	Proximal ileum	Accidental in context of neurodevelopmental delay	Small bowel obstruction, emergency laparotomy with enterotomy and removal of foreign body	Survived
Bakia et al. (2017) [104]	Netherlands	61y F	Plastic bread clip	Small bowel	Accidental	Bread clip ingestion resulted in small bowel perforation and peritonitis, requiring laparotomy and resection of the affected segment	Survived
Mitsui et al. (2018) [105]	Japan	47y M	plastic bread clip	Jejunum	Accidental	Small intestinal perforation (jejunum) with peritonitis and ascites; CT showing clip in jejunum. Emergency laparotomy with resection. Post-op tracheotomy for aspiration pneumonia (POD 3)	Survived; discharged on POD 16
Kim et al. (2013) [106]	South Korea	6y F	lollipop stick	Duodenum	Accidental	lollipop stick perforating the second portion of the duodenum and evidence of peritonitis, liver injury, Laparoscopic extraction of 8 cm lollipop stick via duodenal perforation, Single-layer duodenal closure, Intraoperative OGD, Peritoneal lavage and closed drain placement	Survived, Discharged POD 10
Tawfik et al. (2015) [107]	US	13y F	Disinfection caps	Oesophagus	Accidental	Flexible fibreoptic laryngoscopy identified a foreign body at the cricopharyngeus. Oesophagoscopy with Roth net was used to remove a disinfection cap obstructing the distal oesophagus, which caused a partial-thickness laceration.	Survived
	US	3y M	Disinfection caps	Oesophagus (implied from case report)	Accidental	Had emesis, which ejected the disinfection cap	Survived
Ahn et al. (2011) [108]	South Korea	2y M	Piece of plastic material	Oesophagus	Accidental	Misdiagnosed as asthma; CT and X-ray revealed retained oesophageal plastic foreign body causing tracheoesophageal fistula and tracheal stenosis; surgical removal performed, tracheostomy for persistent tracheal stenosis, full recovery	Survived

*Multiple Objects* Eight (8) case reports of multiple object ingestion were identified, including five (5/8) from a single case series [109-112]. All cases were intentional. Reported anatomical locations included the stomach (2/8), stomach and ileum (1/8), oesophagus and stomach (1/8), hypopharynx (1/8), and bowel segments (3/8: transverse colon 1/8, complete passage through bowel 1/8, unspecified bowel 1/8). One (1/8) death was reported, caused by an aortoesophageal fistula. Demographics were available for all cases: six (6/8) male and two (2/8) female, with a mean age of 22 years.

The following interventions were performed (n=6/8): laparotomy-based (3: laparotomy 2, gastrotomy + laparotomy 1), endoscopy-based (2: with gastrotomy 1, with gastrostomy + thoracotomy 1), intubation + laryngoscopy + bronchoscopy + gastrostomy tube (1).

Table 14 demonstrates case reports of multiple object ingestion.

**Table 14:** Reported cases of multiple object ingestion (eight reported cases from four publications) y - year; M - male; F - female; CT - computed tomograph

Author	Country	Age/ sex	Object	Location	Intent	Interventions/ complications	Outcome
James et al. (1982) [109]	UK	18y M	Darning needle, razor blades	Transverse colon	Intentional	Prior gastrotomy for darning needle. Eleven years later, tissue-wrapped razor blades removed via laparotomy, complicated by faecal fistula. Later ingestion of more blades—likely in transverse colon—passed naturally without complication	Survived
	UK	23y M	Pins, darning needles, pencil, knife, safety pin, pen knife, bed bolt	Stomach	Intentional	Pins removed via gastrotomy after failed endoscopy (complicated by subphrenic abscess); later, darning needles, pencil, knife, and safety pins mostly passed naturally. The penknife was removed surgically. Also swallowed a bed bolt.	Survived
	UK	28y F	Spoons, pins, needles, toothbrush, and stethoscope bell	Bowels	Intentional	Swallowed three spoons. Over 15 years, ingested various objects including pins, needles, toothbrush, and stethoscope bell. Underwent 17 laparotomies for removal with no bowel perforation. Managed conservatively for the past 3 years despite 4 admissions for mild obstruction, resolved with IV fluids and NG decompression.	Survived
	UK	17y M	Nails, razor blades, glass and coins	Stomach	intentional	Presented with haematemesis. Endoscopic removal failed; objects removed via gastrotomy. Developed mediastinal abscesses from an oesophageal tear at the azygos arch, requiring thoracotomy for drainage	Survived
	UK	17y M	Several nuts and bolts	Passed through bowels	intentional	managed conservatively, passed in stools	Survived
Jaroudi et al. (2019) [110]	US	38y M	Plastic spoon and bags	Left hypopharynx	Intentional	Intubated for respiratory distress. Developed subcutaneous and mediastinal emphysema, pneumomediastinum, and pneumopericardium. Microdirect laryngoscopy and rigid bronchoscopy removed a foreign body from the left hypopharynx. IV antibiotics were started due to mediastinitis risk. Repeat laryngoscopy revealed oesophageal dissection from C2 to T2 with posterior wall separation. Supported for healing. Follow-up CT: reduced emphysema, soft tissue thickening, pleural effusions, and pneumonia. After successful extubation, Oesophagram showed contrast in both bronchi, and a gastrostomy tube was placed for nutrition.	Survived
Di Nunno et al. (2000) [111]	Italy	22y M	T-shaped razor, cigarette lighter part, earring, green plastic, fragment of telephone card, 10 oxidized coins (90g)	Oesophagus, stomach and intestines	Intentional	Ingested T-shaped razor lodged in oesophagus, caused aorto-oesophageal fistula. Lighter fragment, earring, plastic piece, 1.4 kg blood found in stomach. Telephone cord fragment, 10 oxidized coins (90 g) lodged near Meckel’s diverticulum. Death from massive aortic hemorrhage due to Aorto-oesophageal fistula - autopsy findings	Death
Dong et al. (2023) [112]	Australia	13y F	Elastic hair ties and headbands	Stomach, ileum	intentional	Exploratory laparotomy uncovered ileal volvulus and a contained small bowel perforation due to a large number of elastic hair ties and headbands in the stomach and small intestine. Segmental ileal resection with re-anastomosis and gastrostomy for foreign body removal	Survived

*Other Objects* Thirteen (13) case reports of various object ingestion were identified, including two (2/13) involving pens [113-125]. Five (5/13) cases were intentional ingestions. Reported anatomical locations included the oesophagus (4/13), oesophagus and aorta (1/13), oesophagus and stomach (2/13), stomach (2/13), stomach and duodenum (2/13), sigmoid colon (1/13), intestines-likely colon (1/13), and a complex multi-site case involving the oral cavity, pharynx, stomach, intestines, bronchus, and airways (1/13). Three (3/13) deaths were reported: one (1/13) from suffocation, one (1/13) from oesophageal-aortic fistula, and one (1/13) from septic shock. Demographic data were available for all thirteen cases: eight (8/13) male and five (5/13) female. Deaths occurred in two (2/8) males and one (1/5) female. Age was reported in ten (10/13) cases (excluding one neonate), with a mean age of 44.5 years.

The following interventions were performed (n=10/13): endoscopy-based (7: alone 4, with transoral video approach 1, with intubation 1, with laryngoscopy + thoracic removal 1), sigmoidoscopy (1), thoracic surgery (1), intubation + endoscopy (counted in endoscopy group above). In three (3/13) cases, no intervention was performed (all fatal).

Table 15 lists all other case reports on ingested objects.

**Table 15:** Reported cases of ingestion of other foreign bodies (13 reported cases from 13 publications) y - year; M - male; F - female; CT - computed tomograph; OGD - oesophago-gastro-duodenoscopy; OGT - orogastric tube

Author	Country	Age/ sex	Object	Location	Intent	Interventions/ complications	Outcome
Riva et al. (2018) [113]	Italy	27y M	Wooden sphere part of necklace	Oesophagus	Intentional (bet)	Failed flexible endoscopic removal; CT: complete upper oesophageal obstruction. Foreign body successfully removed transorally via video-assisted approach	Survived, repeat endoscopy at one month was normal
Maklad et al. (2020) [114]	US	20y M	Stone	Oesophagus at T1-T2	Accidental	Presented with emesis, dysphagia, and shortness of breath; intubated. Multiple failed removal attempts. Foreign body pushed into stomach and removed using combined flexible and rigid Oesophagoscopy.	Survived
Koscove (1987) [115]	US	27y M	Taser dart	Intestines likely colon	Intentional	Conservative management. Day four, dart passed with wire. Asymptomatic, discharged.	Survived
Enon et al. (2007) [116]	Turkey	40y M	Dynamic tracheal stent	Stomach	Accidental	Endoscopic examination showed the stent had migrated into the stomach, successfully retrieved via flexible gastroscopy	Survived
Yocum et al. (2021) [117]	US	Adult female	Multiple crayons	Oesophagus	Accidental	At least 28 crayons were removed over three endoscopic procedures. The patient subsequently developed aspiration pneumonia, respiratory distress, and septic shock	Death
Özdemir et al. (2016) [118]	Turkey	Premature neonate, M	Laryngoscope light bulb	Stomach, then duodenum during USS	Accidental	passed spontaneously wit conservative management	Survived
Patel et al. (2024) [119]	US	65y F	Mussel shell	Oesophagus	Accidental	Upper endoscopy revealed a large piece of mussel shell deeply embedded across opposing walls of the distal Oesophagus, causing two deep linear mucosal tears. Dura-clips applied.	Survived
Suhail et al. (2024) [120]	US	44y M	Pen	Stomach	intentional	Gastric perforation, gastropleural fistula, pleural abscess, Endoscopic snare removal of pen, APC (argon plasma coagulation), over-the-scope (OTS) padlock clip, planned for follow-up elective OGD	Survived, diet advanced 48 hours
Bussell et al. (2021) [121]	US	57y F	Three pens	Oesophagus, stomach, mediastinum	Intentional	Oropharyngeal ulceration, Oesophageal perforation, pneumomediastinum, Direct laryngoscopy removal of first pen, OGD removal of second pen, Thoracic surgical removal of third pen	Survived, discharged seven days later
Tierney et al. (2021) [122]	US	76y F	4cm linear bone fragment	Sigmoid colon	Accidental	A sharp bone fragment became embedded in the sigmoid colonic wall with local inflammation and potential perforation; Sigmoidoscopy retrieved a 4 cm linear bone fragment embedded in the sigmoid wall using rat‑tooth forceps	Survived, pain resolved within 24 hours
Dermircan et al. (2008) [123]	Turkey	63y M	Bone	Oesophagus and aorta	Accidental	Haemoptysis, hematemesis, CT showed a bone in the subcarinal space with mediastinal air. Intraoperatively, it was found to have perforated the oesophagus and aorta, causing an aortoesophageal fistula and fatal bleeding. This case highlights Chiari's triad—midthoracic pain, sentinel bleed, and exsanguination—seen in aortoesophageal fistula from bone ingestion.	Death despite surgery following a bone-induced aortoesophageal fistula
Klein et al. (2014) [124]	Germany	Middle-aged male	Multiple wooden objects	Oral cavity, pharynx, Oesophagus, stomach, intestine, bronchus, and airways	Intentional suicide	Multiple wooden foreign bodies were identified in the oral cavity, pharynx, oesophagus, stomach, intestine, bronchus, and airways, with associated perforation, aspiration, and soft tissue infiltration	Autopsy revealed death from suffocation, airway obstruction, aspiration of wooden foreign bodies, soft tissue injury, pharyngeal and neck perforation
Saeed et al. (2020) [125]	US	26y F	Orogastric tube	Stomach and duodenum	Accidental - iatrogenic	16 Fr Salem-Lump OGT placed for nutrition/medications. Post-hypoxic myoclonus occurred. OGT appeared dislodged; new tube inserted. CXR showed second tube paralleling new OGT. EGD urgently removed transected 44 cm distal OGT from gastric antrum traversing pylorus using rat-tooth forceps.	removed successfully, patient was Post-cardiac arrest

CT and Interventions

Overall, CT imaging was reported to have been performed in 66 cases (47.8%), while in 72 cases (52.2%) its use was not mentioned.

CT demonstrated a distinct detection pattern between fish bones and toothpicks. Fish bones, being partially calcified and radiopaque, were identified in 12 of 16 scans (75%), whereas toothpicks, composed of radiolucent organic wood, were detected in only 3 of 12 scans (25%). Fish bones typically appeared as linear or needle-shaped high-density structures, clearly outlined against soft tissue or within abscess cavities, while toothpicks were seldom visualised.

Although both foreign bodies caused similar complications, such as localised perforation and abscess formation, CT showed markedly greater sensitivity for calcified or mineralised materials than for organic wood. Clinically, the absence of a visible foreign body on CT should not exclude the diagnosis, particularly when a wooden object is suspected, and alternative imaging or endoscopic evaluation may be warranted.

Out of 138 cases, 21 reported no intervention, while the remaining cases accounted for 176 documented procedures. As most foreign bodies were located in the upper gastrointestinal tract, particularly the oesophagus, endoscopy was the most frequently reported procedure, aligning with these findings. In the pooled dataset of 176 performed procedures, the most common interventions were endoscopy (58, 33.0%) and laparotomy (34, 19.3%), followed by laryngoscopy (7, 4.0%), intubation (7, 4.0%), colonoscopy (6, 3.4%), gastrotomy (6, 3.4%), laparoscopy (5, 2.8%), thoracotomy (5, 2.8%), intensive treatment unit/critical care (5, 2.8%), tracheostomy (4, 2.3%), bronchoscopy (3, 1.7%), thoracoscopy/Video-assisted thoracoscopic surgery (2, 1.1%), thoracic endovascular aortic repair (2, 1.1%), and surgical drainage (2, 1.1%).

All other procedures were reported only once each (n=29, 16.5%) and included abscess drainage, angioplasty, carotid ligation, cervical incision, colotomy, corpectomy, ENT drainage, extraperitoneal drainage, feeding jejunostomy, incision and drainage, intra-oral surgical removal, laminectomy, median sternotomy, neck exploration and drainage, pouchoscopy, recto-sigmoidoscopy, scanning electron microscope, sigmoidoscopy, spinal fusion, surgical removal, thoracic removal, thoracic surgery, thyrotomy, transcervical oesophagotomy with thoracic oesophageal myotomy, transoral long forceps, transoral incision, transoral video approach, vascular intervention, and vessel ligation.

There was no statistical analysis performed for this systematic review, as results were qualitative in nature using case studies.

Discussion

We have systematically searched the literature and found a number of useful clinical patterns resulting from foreign body ingestion. It is striking how many serious injuries occur from toothpick and fishbone ingestion; this is likely to be compounded by the relative radiolucency of these objects, limiting the value of radiology. There were multiple harms caused by needles, but we did not find any cases of harm related to drawing pins or safety pins, despite seeing these regularly in our practice. We suggest that the design of these pins prevents harm.

Medication blister packs caused many injuries, and these were exclusively in the elderly. It was unsurprising that larger objects caused harm in the oesophagus and that children, with relatively smaller anatomy, suffered more oesophageal harm. Intentional ingestions were larger and included multiple objects, with a younger age demographic.

There is also a note of caution that many injuries seemed to occur distal to the oesophagus and stomach. Many clinicians will discharge people who have radiographic evidence that the foreign body has passed below the diaphragm. Our results suggest that these patients need safety netting advice, as harm has been described.

Some of our results have been reproduced by other research. We found that toothpicks predominantly affected the sigmoid colon, frequently causing perforation. Toothpicks had poor CT identification rates. All three of these findings are reproduced in the Li et al. study [126]. Fish bones had multiple complications and interventions described, with another systematic review supporting these findings and suggesting early intervention is required [127]. This bolsters the reliability of this review.

Our findings regarding paediatric coin ingestion are reproduced in another systematic review by Jayachandra et al., which demonstrates that harm usually occurs within the first one-third of the oesophagus, in keeping with our findings [128]. 

Our results suggest that ingestions of safety pins and drawing pins are unlikely to result in harm, as there were no publications including these within our search.

Many foreign body ingestions did not have existing systematic reviews, including chicken bones and blister packs, to compare with. The literature on chicken bone and blister pack ingestion is case report-based; therefore included within our review, so not directly comparable. 

Incidence of mortality in self-harm foreign body ingestion was found to be 6.66% in a separate study, with post-operative complications in 22.2% [129]. Our study found a higher rate of mortality at 20% with intentional (self-harm) ingestions. This may be due to publication bias favouring outcomes involving complications or mortality.

Limitations

Our methodology does not allow us to describe the epidemiology or quantify the harm of foreign body ingestion, as case reports do not provide denominators. Our results are also prone to reporting biases; unusual foreign bodies and injuries are more likely to be published. Furthermore, publication of harm caused by intentional ingestion is likely to be underreported, as patients are less likely to consent to their information being used.

There is no validated or widely accepted tool for assessing bias in systematic reviews of case reports; therefore, one was not used for this systematic review. There is currently no validated tool used to assess the bias of case reports. Case reports are regarded as a relatively weak form of evidence, and the risk of bias is high, especially for publication bias.

We did not include grey literature or non-peer-reviewed journals. This is an issue because peer-reviewed journals are more likely to publish studies with statistically significant or positive results. Consequently, excluding grey literature, this review may overestimate effects or miss null/negative findings, skewing conclusions.

Our results did not include magnets, batteries or poisonings, and are therefore not generalisable to these foreign body ingestions.

Strengths

Our search strategy is robust and inclusive, covering multiple medical databases and a deliberately inclusive strategy. This is the first systematic review that covers all foreign body ingestions; previously, these have been restricted to children [128], intentional ingestion [130] or to upper gastrointestinal harm [131]. Our results are more useful, as an emergency physician may not know where in the GI tract harm is likely to occur when they evaluate a patient after ingestion. 

Implications for clinical practice

Patients who present after ingestion of toothpicks, fish bones, or needles may require extended observation, even if initially stable. CT is the most useful imaging modality, but it is not infallible; radiolucent objects are frequently missed, and harm may occur despite apparently reassuring scans. Importantly, patients who appear well or whose objects have passed beyond the diaphragm must still receive robust safety-netting advice, as delayed complications have been well documented. No features were identified that reliably confer freedom from harm.

Our findings neither contradict nor support existing guidelines, e.g., European Society of Gastrointestinal Endoscopy (ESGE) guidelines [132], which are inevitably based on low-quality evidence and are more concerned with which patients need an endoscopy and the endoscopic techniques used. Our findings illustrate the serious harms that can result, especially from long and sharp objects, and should influence discharge advice with heavy emphasis on robust safety netting for patients to return to the hospital early if they become symptomatic of nausea, vomiting or abdominal pain.

Future research directions could focus on identifying patients who should have clinical intervention in the form of endoscopy or surgery early, even if the foreign body has passed beyond the diaphragm. Most guidelines focus on the use of endoscopy, but there is are no robust guidelines for how to manage foreign body ingestion once foreign bodies have passed beyond the diaphragm.

## Conclusions

Ingested foreign bodies cause significant morbidity and mortality across both adults and children, with harm documented at multiple gastrointestinal sites. Distinct clinical patterns emerged: children were more likely to sustain proximal oesophageal injury from coins, while adults experienced distal complications from sharp or elongated objects such as toothpicks, bones, and dental appliances. Blister packs were reported almost exclusively in elderly patients, whereas intentional ingestions clustered in younger individuals with psychiatric comorbidity.

Mortality did not differ substantially between accidental and intentional ingestion (19.6% vs 20.0%), but object type strongly influenced outcome. Chicken bones (50%), fish bones (28%), coins (38%, primarily in children), and toothpicks (18%) carried the highest case fatality. These findings reinforce that age and object type together provide the best guide to risk stratification.

Computed tomography was the most useful imaging modality and was frequently employed, yet it was not infallible. Radiolucent objects such as toothpicks and fish bones were often missed, and several patients developed severe complications despite apparently reassuring imaging or distal passage of the object. A negative scan, therefore, does not exclude risk.

Safe management requires clinicians to integrate knowledge of object-specific harm patterns, patient age, and intent into their decision-making. Patients who are clinically well at presentation but have ingested high-risk objects should only be discharged with robust safety-netting advice and clear follow-up arrangements. Careful consideration of both the object ingested and its likely natural history is essential to prevent delayed morbidity and mortality.

## Appendices

**Table 16:** Ovid MEDLINE/PudMed Search 1946 to June 24, 2025.

Step	Search Terms	Results
1	Foreign Bodies/	31,609
2	(Foreign bod* or foreign object* or retained foreign material or non-native object* or inserted object* or iatrogenic object* or implanted object* or foreign matter).ti,ab.	41,276
3	1 or 2	56,604
4	Deglutition/	12,557
5	(Ingest* or swallow*).ti,ab.	162,268
6	4 or 5	165,277
7	Death/	20,951
8	(Harm or adverse event* or iatrogenic or drug* or adverse effect* or death or disability).ti,ab.	3,633,176
9	7 or 8	3,641,622
10	3 and 6 and 9	568
11	limit 10 to (english language and humans)	353

**Table 17:** CINAHL/Ebsco search 1995 to June 22, 2025.

Step	Search Terms	Results
1	(MH "Foreign Bodies")	6,085
2	XB (Foreign bod* or foreign object* or retained foreign material or non-native object* or inserted object* or iatrogenic object* or implanted object* or foreign matter)	5,956
3	1 OR 2	9,338
4	(MH "Deglutition")	4,513
5	XB (Ingest* or swallow*)	28,749
6	4 OR 5	29,808
7	XB (Harm or adverse event* or iatrogenic or drug* or adverse effect* or death or disability)	777,926
8	(MH "Death")	8,148
9	(MH "Adverse Health Care Event")	5,603
10	7 OR 8 OR 9	781,940
11	3 AND 6 AND 10	121
12	3 AND 6 AND 10 (Humans and English Language only)	10

**Table 18:** Ebsco search 1974 to June 21, 2025.

Step	Search Terms	Results
1	foreign body/	42,929
2	(Foreign bod* or foreign object* or retained foreign material or non-native object* or inserted object* or iatrogenic object* or implanted object* or foreign matter).ti,ab.	48,094
3	1 or 2	66,381
4	swallowing/	32,122
5	(Ingest* or swallow*).ti,ab.	222,678
6	4 or 5	228,429
7	adverse outcome/	78,714
8	death/	297,897
9	(Harm or adverse event* or iatrogenic or drug* or adverse effect* or death or disability).ti,ab.	5,278,124
10	7 or 8 or 9	5,401,201
11	3 and 6 and 10	861
12	limit 11 to (human and english language)	719
